# Patient-reported outcomes in randomised clinical trials of bladder cancer: an updated systematic review

**DOI:** 10.1186/s12894-019-0518-9

**Published:** 2019-09-14

**Authors:** Mieke Van Hemelrijck, Francesco Sparano, Debra Josephs, Mirjam Sprangers, Francesco Cottone, Fabio Efficace

**Affiliations:** 1 King’s College London, School of Cancer and Pharmaceutical Sciences, Translational Oncology and Urology Research (TOUR), London, SE1 9RT UK; 2 Data Center and Health Outcomes Research Unit, Italian Group for Adult Hematologic Disease (GIMEMA), Rome, Italy; 3 Guy’s and St Thomas’ NHS Foundation Trust, Medical Oncology, London, UK; 4 Department of Medical Psychology, Location AMC, Amsterdam University Medical Centers, Amsterdam, The Netherlands

**Keywords:** PROs, Quality of life, Outcome measurement, RCT, Bladder cancer

## Abstract

**Background:**

Despite international recommendations of including patient-reported outcomes (PROs) in randomised clinical trials (RCTs), a 2014 review concluded that few RCTs of bladder cancer (BC) report PRO as an outcome. We therefore aimed to update the 2014 review to synthesise current evidence-based knowledge of PROs from RCTs in BC. A secondary objective was to examine whether quality of PRO reporting has improved over time and to provide evidence-based recommendations for future studies in this area.

**Methods:**

We conducted a systematic literature search using PubMed/Medline, from April 2014 until June 2018. We included the RCTs identified in the previous review as well as newly published RCTs. Studies were evaluated using a predefined electronic-data extraction form that included information on basic trial demographics, clinical and PRO characteristics and standards of PRO reporting based on recommendation from the International Society of Quality of Life Research.

**Results:**

Since April 2014 only eight new RCTs for BC included PROs as a secondary outcome. In terms of methodology, only the proportion of RCTs documenting the extent of missing PRO data (75% vs 11.1%, *p* = 0.03) and the identification of PROs in trial protocols (50% vs 0%, *p* = 0.015) improved. Statistical approaches for dealing with missing data were not reported in most new studies (75%).

**Conclusion:**

Little improvement into the uptake and assessment of PRO as an outcome in RCTs for BC has been made during recent years. Given the increase in (immunotherapy) drug trials with a potential for severe adverse events, there is urgent need to adopt the recommendations and standards available for PRO use in bladder cancer RCTs.

## Background

With an estimated 549,000 new cases and 200,000 deaths in 2018 worldwide, bladder cancer is the 10th most common form of cancer [1]. All groups of bladder cancer patients are, not surprisingly, subjected to significant treatment burdens that are emotionally and psychologically taxing. Several symptoms, such as blood in the urine, pain and nausea, associated with different treatments may result in increased prevalence of depression, anxiety and stress and, consequently, decreased quality of life (QoL) [2]. Given this disease burden, there is a need to further evaluate how patient-reported outcomes (PROs) are incorporated in clinical bladder cancer research. Inclusion of QoL or other PROs in clinical trials and methodological rigor already at the stage of protocol writing are essential to eventually generate data that can robustly inform patient care [3].

Randomized controlled trials (RCTs), across a wide range of cancer malignancies, increasingly include PROs in an effort to better understand overall treatment effectiveness of newer drugs [4]. Inclusion of PROs in cancer research is not only valued by oncologists and patients, but also by regulatory stakeholders. To illustrate, the US Food and Drug Administration (FDA) included PROs as one of the clinical outcomes assessments (COAs) that can be used to determine whether or not a drug has demonstrated treatment benefit [5]. Similarly, the European Medicines Agency (EMA) has issued recent guidelines on the use of PRO endpoints in cancer research [6].

In the context of bladder cancer, a systematic review encompassing the years 2004–2014 examined the quality of PRO reporting and methodological strengths and weaknesses of RCTs. It concluded that few RCTs report PRO as an outcome and improvement in methodology was required [7]. Another more recent systematic review using the COnsensus-based Standards for the selection of health Measurement INstruments (COSMIN) [8] specifically evaluated the psychometric properties of PRO measurements in bladder cancer (1990–2017) [9]. No existing PRO stood out as the most appropriate to measure QoL in bladder cancer patients due to heterogeneity of the disease and its treatments and due to lack of validation studies [9]. Moreover, a recent systematic review highlighted the mental health implications in bladder cancer patients [10] – and hence the potential effects the disease and its treatments can have on QoL.

This study therefore aimed to update the review by Feuerstein et al. [7], by including all the RCTs of that review as well as newly published RCTs in order to synthesise current evidence-based knowledge of PROs from RCTs in bladder cancer. A secondary objective was to examine whether quality of PRO reporting improved over time and to provide evidence-based recommendations for future studies in this area.

## Methods

### Search strategy and identification of studies

We conducted a systematic literature search using PubMed/Medline, from April 2014 until June 2018. Methodology for study identification and evaluation followed standardised criteria used in the PROMOTION Registry (http://promotion.gimema.it) and was previously described in similar systematic reviews [7, 11, 12]. For the purpose of this updated review on bladder cancer RCTs, the following script was used to identify a PRO component: *(“quality of life” OR “health related quality of life” OR “health status” OR “health outcomes” OR “patient outcomes” OR “depression” OR “anxiety” OR “emotional” OR “social” OR “psychosocial” OR “psychological” OR “distress” OR “social functioning” OR “social wellbeing” OR “emotional” OR “patient reported symptom” OR “patient reported outcomes” OR pain OR fatigue OR “patient reported outcome” OR “PRO” OR “PROs” OR “HRQL” OR “QOL” OR “HRQOL” OR “symptom distress” OR “symptom burden” OR “symptom assessment” OR “functional status” OR sexual OR functioning) AND bladder*. The search strategy was restricted to RCTs. In case of multiple publications from the same RCT, all relevant data possibly published in secondary articles were combined.

### Selection criteria

Only English-language reports of RCTs comparing conventional treatments and involving adult patients with bladder cancer were included – irrespective of disease stage. The minimum, overall sample size was set at 50 patients. Screening studies or those involving patients with benign disease were excluded. We did not consider conference abstracts as these did not contain sufficient information. RCTs of interventions that were psychological, behavioural, complementary or alternative were also excluded.

We included all studies evaluating a PRO either as a primary or secondary outcome – either as a multidimensional QoL outcome or any other type of PRO. Those studies evaluating only treatment adherence or satisfaction were also excluded. For comparability purposes, selection criteria of eligible articles were the same as of the previous systematic review [7]. Details on the search strategy and selection process were documented according to the PRISMA guidelines [13].

#### Methods of evaluation of studies

Two reviewers (MVH, FS) extracted information from the identified studies and a third reviewer (FE) was consulted in case of disagreement. All data were entered by the reviewers into a password protected online database (REDCap) [14] by completing a predefined electronic-data extraction form (eDEF). Full details on information contained in the eDEF have been previously reported [11]. A double-blind data entry procedure was performed as each reviewer completed the eDEF independently. Discrepancies in evaluations were electronically recorded and when disagreements occurred in the evaluation of any item included in the eDEF, the reviewers revisited the paper to reconcile any differences until consensus was achieved.

#### Type of data extraction and data analysis

For the purpose of this review, the following types of information were considered: 1) basic trial demographics; 2) clinical and PRO characteristics and 3) elements of PRO reporting based on recommendation from the International Society of Quality of Life Research (ISOQOL) [15]. Quality of PRO reporting was therefore evaluated with the ISOQOL checklist, which comprises a common set of 17 key issues regardless of PRO being primary or secondary outcome. Eleven additional issues were considered when a PRO is a primary outcome of the study. Each item of the ISOQOL checklist was rated as ‘yes’ if documented in the publication (scored as 1) or ‘no’ if not documented (scored 0). To further refine the investigation of the accuracy of reporting, we divided the ISOQOL item addressing the problem of missing data into two (i.e., reporting the extent of missing data and reporting statistical approaches for dealing with missing data). We thus rated each RCT with a score ranging from 0 to a maximum of 18 (RCT with PRO as a secondary outcome) or 29 (PRO as primary outcome). In both cases, a higher score indicates better quality of the PRO reporting. Our rule of thumb for this analysis was to consider RCTs addressing less than 50% of items included in the ISOQOL recommendations [15] as having “suboptimal quality”. That is, 9 items out of 18 for RCTs which included PRO as secondary outcome and 15 items out of 29 for RCTs which included PRO as primary outcome.

Main characteristics of eligible studies were reported by proportions, means and standard deviation, according to the type of variable. Differences between studies were assessed by Fisher exact test or Wilcoxon-Mann-Whitney test. Based on the ISOQOL checklist score, comparisons of reporting quality were performed. To ensure comparability between studies with PRO as primary or secondary outcome, for each study the raw score was standardised dividing it by the number of applicable items (18 for secondary or 29 for primary), then multiplied by 100. This way, we obtained an adjusted checklist score ranging from 0 (worst quality) to 100 (best quality). Based on such score, we compared studies with PRO as secondary outcome (studies until March 2014 vs those from April 2014), studies with PRO as primary vs. studies with PRO as secondary outcome and studies using a validated PRO measure or not. In addition, we computed the proportion of studies that had a checklist score below or equal to the cut-off value of 50. All tests were two-sided and statistical significance was set at α = 0.05. Analyses were performed by SAS software v. 9.4 (SAS Institute Inc., Cary, NC).

## Results

### Overview of RCT characteristics

**Fig. 1:**
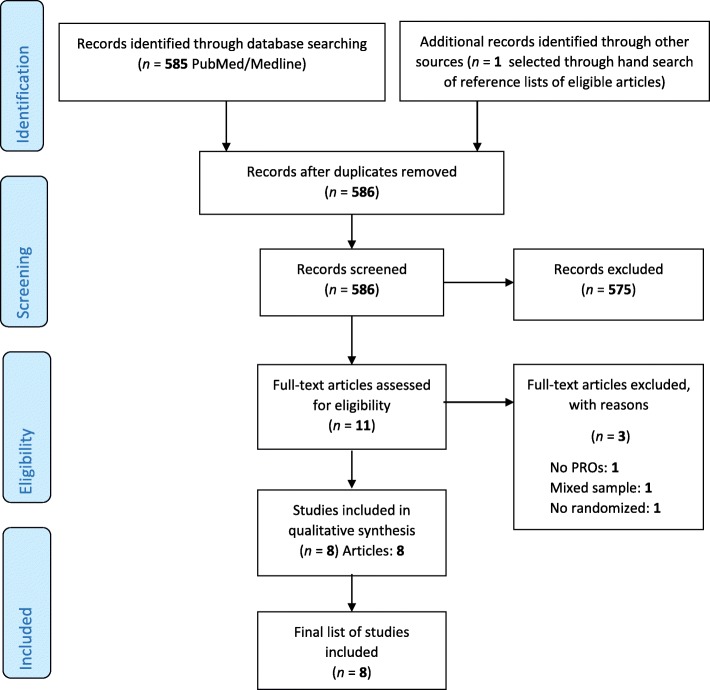
Schematic breakdown of literature search results of Bladder Randomized Controlled Trials (Preferred Reporting Items for Systematic Reviews and Meta-analysis). PRO = patient-reported outcomes

The search identified 586 abstracts published in the period 2014–2018. Eight studies fulfilled the eligibility criteria (Fig. 1). In all the newly identified RCTs [16–23], PROs were secondary outcome, whereas of the nine old studies [24–33] five RCTs (55.6%) employed PROs as primary outcome [27–30, 33]. All but one of the newly identified RCTs were not supported by industry (87.5%) and none of the RCTs was carried out in a multinational context. The majority of new trials (5, 62.5%) enrolled patients with non-metastatic disease. Compared to the old studies, where two RCTs (22.2%) enrolled more than 200 patients, only one of the newly identified RCTs (12.5%) enrolled more than 200 patients overall. Six new RTCs (75%) assessed PROs over a time period of 6 months, one study (12.5%) up to 1 year and in one study (12.5%) the length of assessment was more than 1 year. Details are reported in Table 1.

**Table 1:** Overview of RCT characteristics

Characteristic	Category	RCT published between Jan.2004 –Mar. 2014 (n. 9), N. (%)	RCTs published between Apr.2014 – Jun.2018 (n. 8), N. (%)	Total (n. 17), N. (%)
International (if more than one country)	No	8 (88.9)	8 (100)	16 (94.1)
	Yes	1 (11.1)	0 (0)	1 (5.9)
Industry supported (fully or in part)	No	6 (66.7)	7 (87.5)	13 (76.5)
	Yes	3 (33.3)	1 (12.5)	4 (23.5)
PRO endpoint	Primary	5 (55.6)	0 (0)	5 (29.4)
	Secondary	4 (44.4)	8 (100)	12 (70.6)
Secondary paper on PRO	No	8 (88.9)	8 (100)	16 (94.1)
	Yes	1 (11.1)	0 (0)	1 (5.9)
Length of PRO assessment during RCT	Up to 6 months	4 (44.5)	6 (75)	10 (58.8)
	Up to 1 year	2 (22.2)	1 (12.5)	3 (17.7)
	More than 1 year	3 (33.3)	1 (12.5)	4 (23.5)
Overall study sample size	<=200	7 (77.8)	7 (87.5)	14 (82.3)
	> 200	2 (22.2)	1 (12.5)	3 (17.7)

### Most recent (2014–2018) evidence of bladder cancer RCTs with PROs

Among the eight newly identified RCTs, only three [18, 19, 23] used a multidimensional PRO instrument (e.g. the European Organization for Research and Treatment of Cancer Quality of Life Questionnaire-Core 30 (EORTC QLQ-C30) [34]) and of these, two used a bladder cancer-specific questionnaire (the Functional Assessment of Cancer Therapy-Bladder (FACT-Bl) and the FACT-Vanderbilt Cystectomy Index (VCI) questionnaires) (Table 2). In three studies [16, 17, 20], no differences in pain scores were detected between the experimental treatment arms (solifenacin, sevoflurane and glycopyrrolate, respectively) and the control arms (standard care, desflurane and atropine, respectively). In the study conducted by Huang et al. [21], VAS scores for bladder pain were significantly lower at the end of the induction cycle in the group treated with pirarubicin combined with hyaluronic acid compared to pirarubicin alone, while dexmedetomidine was associated with lower postoperative pain scores compared to placebo [22]. Low dose of Bacillus Calmette-Guerin (BCG) was associated with better outcomes in terms of global QoL, role functioning and financial problems, as assessed by the EORTC QLQ-C30, compared to standard dose [18]. No differences in QoL, as assessed with the FACT-BI, were found between laparoscopic and robot-assisted radical cystectomy [19]. Finally, no difference in QoL, as assessed by the FACT-VCI, was detected between robot-assisted radical cystectomy and open radical cystectomy [23].

**Table 2:** Overview of bladder cancer RCTs with a PRO evaluation published between 2004 and 2018

Author	Intervention	Type of bladder cancer	Sample size^a^	Main Clinical Outcome	PRO instrument used	Summary findings for main clinical outcome and PRO^b^
PRO primary endpoint						
Marandola et al. 2005 [27]	Spinal anaesthesia with 10 mg of 0.5% hyperbaric bupivacine vs. 15 μg of sufentanil	Scheduled for TURBT	62	Motor and sensory blockages (primary)	Verbal analogue pain scale	• Bupivacine patients experienced more intense motor blockade • Statistical significance on PRO outcomes not reported
Ozyuvaci et al. 2005 [28]	General anaesthesia vs combined epidural and general anaesthesia for radical cystectomy	Scheduled for radical cystectomy	50	Intraoperative outcomes	Visual analogue scale	• Significant reduction of intraoperative blood loss for those in the combined group • Lower post-operative pain scores for the combined group
Gontero et al. 2013 [29]	Intravesical gemcitabine vs 1/3 dose BCG instillation	NMIBC	120	Recurrence and progression	EORT QLQ-C30	• No difference in recurrence and progression • On univariate analysis, at T1, gemcitabine had better cognitive and emotional functioning and urinary symptom distress. At T2, gemcitabine had better cognitive functioning and less nausea and vomiting symptom distress
Johnson et al. 2013 [33]	10 mg extended release oxybutynin daily or placebo 6 weeks prior to BCG treatment	BCG Naïve NMIBC	50	Adverse reactions and systemic symptoms	Self-reported urinary symptoms	• More urinary frequency and burning, fever and flu-like symptoms when receiving treatment • Worse urinary symptoms when receiving treatment
Karl et al. 2014 [30]	Early recovery vs conservative regimen after radical cystectomy	Scheduled for radical cystectomy	101	Postoperative morbidity, adverse events, mobility	EORTC QLQ-C30	• Early recovery associated with lower rates of wound healing disorders, DVT and fever • Early recovery associated with improvements in most QLQ-C30 scales
PRO secondary endpoint						
Skinner et al. 2009 [31] Ahmadi et al. 2013 [32]	T pouch vs Studer pouch diversion after radical cystoprostatectomy	Scheduled for cystoprostatectomy	295	Renal function and anatomy at 3 years following surgery (primary)	Modified version of the Bladder Cancer Index	• No differences • Not reported
Koga et al. 2010 [26]	Maintenance vs observation following complete response after BCG	NMIBC	53	Efficacy of duration (primary)	EORTC QLQ-C30	• Maintenance BCG associated with lower recurrence rate on univariate, but not multivariate, analyses • No difference in QoL
Sabichi et al. 2011 [25]	Celecoxib vs. placebo	NMIBC	146	Time to recurrence (primary)	EORTC QLQ-C30	• No effect on time to recurrence. • No difference in QoL
James et al. 2012 [24]	Radiotherapy with or without chemotherapy	MIBC	360	Survival free of locoregional disease (primary)	Not reported	• Locoregional disease-free survival was significantly better in the chemoradiotherapy group than in the radiotherapy group • PRO not reported
Kim et al. 2015 [20]	Glycopyrrolate vs atropine in combination with neostigmine after TURBT	Scheduled for TURBT	74	Incidence of catheter-related bladder discomfort postoperatively (primary)	Numerical rating scale	• Incidence of CRBD was significantly lower in the glycopyrrolate group than in the atropine group postoperatively • No difference in pain scores
Huang et al. 2015 [21]	Pirarubicin combined with hyaluronic acid vs pirarubicin alone after TURBT	Scheduled for TURBT	127	Recurrence (efficacy) (primary)	Visual analogue scale	• No difference in treatment efficacy • The VAS for bladder pain was significantly lower, at the end of the induction cycle, in the experimental group
Kim et al. 2015 [22]	Dexmedetomidine vs placebo during TURBT	Scheduled for TURBT	109	Incidence of catheter-related bladder discomfort postoperatively (primary)	Numerical rating scale	• Incidence of CRBD was significantly higher in the control group • The postoperative pain score was higher in the control group
Kim et al. 2016 [17]	Sevoflurane vs desflurane during TURBT	Scheduled for TURBT	89	Incidence of catheter-related bladder discomfort 24 h postoperatively (primary)	Numerical rating scale	• Sevoflurane was associated with less frequent postoperative CRBD • No difference in postoperative pain scores
Yokomizo et al. 2016 [18]	80 mg BCG (standard) vs 40 mg BCG induction therapy	NMIBC or CIS	166	Non-inferiority with a null hypothesis of 15% decrease in complete response rate	EORT QLQ-C30	• Noninferiority not proven. • Low dose BCG associated with higher quality of life
Khan et al. 2016 [19]	Laparoscopic radical cystectomy vs. robot-assisted radical cystectomy	Scheduled for cystectomy	60	30- and 90-day complication rates	FACT-Bl	• 30-d complication rate higher in the open radical prostatectomy arm; but no differences at 90-d • No difference in QoL between both arms
Chung et al. 2017 [16]	Solifenacin vs standard care prior, during, and after TURBT	NMIBC	134	Incidence of catheter-related bladder discomfort (CRBD) at 1 and 2 h post TURBT (primary)	Visual analogue scale	• No difference in incidence rates • No difference in postoperative pain scores
Parekh et al. 2018 [23]	Robot-assisted radical cystectomy vs. open radical cystectomy	Scheduled for cystectomy	302	Progression-free survival at 2 years after surgery	Short Form-8 FACT-VCI	• Robotic cystectomy was non-inferior to open cystectomy for 2 years progression-free survival • No difference in QoL between both arms

*Abbreviations*: *BCG* Bacillus Calmette-Guerin, *CIS* Carcinoma in situ, *CRBD* Catheter-related bladder discomfort, *DVT* Deep vein thrombosis, *EORTC* European Organization for Research and Treatment of Cancer, *FACT-BI* Functional Assessment of Cancer Therapy-Bladder, *FACT-VCI* Functional Assessment of Cancer Therapy-Vanderbilt Cystectomy Index, *MIBC* Muscle invasive bladder cancer, *NMIBC* Non-muscle invasive bladder cancer, *PRO* Patient-Reported Outcomes, *QLQ-C30* Quality of Life Questionnaire-Core30, *QoL* Quality of life, *RCT* Randomized controlled trial, *TURBT* Transurethral resection of bladder tumor

^a^The overall trial sample size refers to all the patients that agreed to participate to the study giving informed consent. We refer to the number of patients actually enrolled, not necessarily those who were randomized

^b^Differences in the main traditional clinical outcome were extracted based on reported statistical significance. Differences in PRO outcomes were based on statistical significance and/or clinically meaningful difference

### Comparison of PRO quality reporting between 2004 and 2014 and 2014–2018

Only one (12.5%) of the eight new RCTs reported a PRO hypothesis [18] and two (25%) reported the statistical approach for dealing with missing data [19, 23]. Three RCTs (37.5%) documented the mode of PRO administration [16, 17, 21], four (50%) documented the rationale for the choice of PRO instrument [16, 19, 21, 23], whereas two RCTs (25%) reported generalisability issues [17, 21] or interpretation in terms of clinical significance [21, 22].

Compared to previous studies, only two statistically significant improvements were noted: there was an increase in proportion of RCTs documenting the extent of missing PRO data (75% vs 11.1%, *p* = 0.015) and an increase of RCTs documenting PROs in trial protocols (50% vs 0%, *p* = 0.03). Further details are reported in Table 3.

**Table 3:** Comparison of PRO quality reporting over time in Bladder Cancer RCTs with PROs as a secondary outcome

Methodological issue	Category	RCT with PROs (Jan.2004 –Mar. 2014) (n. 9), N. (%)	RCTs with PROs (Apr.2014 – Jun.2018) (n. 8), N. (%)	*P*-value
Title and abstract				
The PRO should be identified as an outcome in the abstract	No	1 (11.1)	3 (37.5)	0.29
	Yes	8 (88.9)	5 (62.5)	
Introduction, background, and objectives				
The PRO hypothesis should be stated and specify the relevant PRO domain if applicable	No	5 (55.6)	5 (62.5)	1
	Yes	2 (22.2)	1 (12.5)	
	N/A (if explorative)	2 (22.2)	2 (25)	
Methods				
*Outcomes*				
The mode of administration of the PRO tool and the methods of collecting data should be described	No	7 (77.8)	5 (62.5)	0.62
	Yes	2 (22.2)	3 (37.5)	
Electronic mode of PRO administration^a^	No	1 (11.1)	2 (25)	1
	Yes	1 (11.1)	0 (0)	
	N/A	7 (77.8)	6 (75)	
The rationale for choice of the PRO instrument used should be provided	No	4 (44.4)	4 (50)	1
	Yes	5 (55.6)	4 (50)	
Evidence of PRO instrument validity and reliability should be provided or cited	No	4 (44.4)	3 (37.5)	0.44
	Yes, for all PRO instruments	5 (55.6)	3 (37.5)	
	Yes, only for some PRO instruments	0 (0)	2 (25)	
The intended PRO data collection schedule should be provided	No	2 (22.2)	1 (12.5)	1
	Yes	7 (77.8)	7 (87.5)	
PROs should be identified in the trial protocol post-hoc analyses	No	9 (100)	4 (50)	0.03^a^
	Yes	0 (0)	4 (50)	
The status of PRO as either a primary or secondary outcome should be stated	No	2 (22.2)	3 (37.5)	0.62
	Yes	7 (77.8)	5 (62.5)	
*Statistical methods*				
There should be evidence of appropriate statistical analysis and tests of statistical significance for each PRO hypothesis tested	No	0 (0)	2 (25)	0.223
	Yes	2 (22.2)	0 (0)	
	N/A	7 (77.8)	6 (75)	
The extent of missing data should be stated^b^	No	8 (88.9)	2 (25)	0.015^a^
	Yes	1 (11.1)	6 (75)	
Statistical approaches for dealing with missing data should be explicitly stated^b^	No	9 (100)	6 (75)	0.206
	Yes	0 (0)	2 (25)	
Results				
*Participant flow*				
A flow diagram or a description of the allocation of participants and those lost to follow-up should be provided for PROs specifically	No	7 (77.8)	5 (62.5)	0.62
	Yes	2 (22.2)	3 (37.5)	
The reasons for missing data should be explained	No	8 (88.9)	5 (62.5)	0.294
	Yes	1 (11.1)	3 (37.5)	
*Baseline data*				
The study patients characteristics should be described including baseline PRO scores	No	6 (66.7)	3 (37.5)	0.347
	Yes	3 (33.3)	5 (62.5)	
*Outcomes and estimation*				
PRO outcomes also reported in a graphical format^a^	No	5 (55.6)	6 (75)	0.62
	Yes	4 (44.4)	2 (25)	
Discussion				
*Limitations*				
The limitations of the PRO components of the trial should be explicitly discussed	No	5 (55.6)	4 (50)	1
	Yes	4 (44.4)	4 (50)	
*Generalizability*				
Generalizability issues uniquely related to the PRO results should be discussed	No	5 (55.6)	6 (75)	0.62
	Yes	4 (44.4)	2 (25)	
*Interpretation*				
PROs are interpreted (Not only re-stated)^a^	No	2 (22.2)	5 (62.5)	0.153
	Yes	7 (77.8)	3 (37.5)	
The clinical significance of the PRO findings should be discussed	No	6 (66.7)	6 (75)	1
	Yes	3 (33.3)	2 (25)	
Methodology used to assess clinical significance is discussed^a^	Anchor based (e.g., minimal important difference)	1 (11.1)	0 (0)	1
	Distribution based (e.g. effect size)	1 (11.1)	2 (25)	
	Both	1 (11.1)	0 (0)	
	Missing	6 (66.7)	6 (75)	
The PRO results should be discussed in the context of the other clinical trial outcomes	No	2 (22.2)	1 (12.5)	1
	Yes	7 (77.8)	7 (87.5)	

For descriptive purposes, subheadings of this table reflect those reported in the ISOQOL recommended standards [15]; however, rating of items was independent of location of the information within the manuscript

^a^These items were not included in the ISOQOL recommended standards [15] and in the calculation of the ISOQOL score but have been evaluated in our study and reported in this table to have a wider outlook on the level of reporting

^b^These items were originally combined in the ISOQOL recommended standards [15] but have been split in this report to better investigate possible discrepancies between documentation of PRO missing data (ie, reporting how many patients did not complete a given questionnaire at any given time point) versus actual reporting of statistical methods to address this issue. Also, we wanted to be consistent with items reported in the CONSORT PRO Extension [35] (ie, statistical approaches for dealing with missing data is reported as a stand-alone issue)

We compared the ISOQOL scores for studies with a PRO as secondary outcome identified in the previous review with those identified in this update. The quality of PRO reporting was considered as “suboptimal” for all of the old studies, while this was not the case for the new RCTs, whose quality was considered suboptimal in 50% of the studies. The mean standardized score for the old studies was 30.5 (median 33.3), while for the new studies the mean score was 48.6 (median 50). However, this positive trend was not statistically significant (*p* = 0.072).

The quality of PRO reporting among all of the studies published between 2004 and 2018 was found to be poor. Overall, only six studies (35.3%) addressed 50% or more of the issues recommended by the ISOQOL checklist (data not shown). The mean standardised ISOQOL score for all these studies was 44.7, below the cut-off value of 50. For three of the five RCTs with PRO as primary outcome (60%) the quality of PRO reporting was considered as “suboptimal”. This percentage was higher for RCTs with a PRO as secondary outcomes, with eight of the twelve studies (66.6%) considered as “suboptimal”. The mean standardised ISOQOL score for the RCTs with PRO as primary outcome was 49.7, while for RCTs with PRO as secondary outcome was 42.6. No statistically significant differences in the ISOQOL score were found between RCTs with PRO as primary or secondary outcomes (*p* = 0.459).

It needs to be noted that only one of the seven (14.3%) studies using validated PRO instruments (e.g. EORTC QLQ-C30) had a high level of quality of PRO reporting, compared to those using non-validated instruments (5 RCTs, 50%). No differences were found in the mean standardised ISOQOL scores between the studies that used validated PRO instruments and those using non-validated instruments.

## Discussion

Since April 2014 only eight new RCTs for bladder cancer that also included a PRO component, were identified and in all these studies PROs were considered as secondary outcomes. Also, during this time period little improvements were noted in the quality of PRO reporting. Indeed, when comparing the new studies identified in this update with previously published RCTs between January 2004 and March 2014 [7], we did not find significant improvement in the mean standardised ISOQOL checklist scores, possibly due to the small number of studies considered. When comparing each individual item of the ISOQOL checklist over time, we only observed two statistically significant improvements with respect to the reporting of missing data and the identification of PROs in trial protocols. Some of the key recommended issues (e.g. reporting of statistical approaches for dealing with missing data, PRO hypothesis statement and generalizability issues regarding the PRO results) are still poorly documented.

The number of newly conducted RCTs of bladder cancer with a PRO component published from 2014 is strikingly low when compared with the number of RCTs conducted in other cancer types, such as breast, lung and prostate cancer [4, 36, 37]. Nevertheless, in the current era of immunotherapy development, including monoclonal antibodies directed against inhibitory checkpoints receptors on T-cells (known as immune checkpoint inhibitors, ICIs), a vast number of trials for bladder cancer are under way – with several of them also assessing PROs. For instance, CHECKMATE 274 (ClinicalTrials.gov Identifier: NCT02632409) is an RCT of the ICI nivolumab versus placebo in patients who have undergone radical cystectomy for muscle-invasive bladder cancer (MIBC). In this study PROs are evaluated as an exploratory outcome using a multidimensional QoL measure. Another study of an ICI, avelumab, in the maintenance setting following first line chemotherapy (JAVELIN; ClinicalTrials.gov Identifier: NCT02603432) also evaluates PRO as a secondary outcome. POTOMAC (ClinicalTrials.gov Identifier: NCT03528694), a trial of the ICI durvalumab plus Bacillus Calmette-Guerin (BCG) versus BCG alone in patients with high risk non-muscle invasive bladder cancer (NMIBC) assesses several PROs as secondary outcome measures. Of note however, many currently ongoing studies in bladder cancer, including those evaluating PARP-inhibitors, FGFR-inhibitors and tyrosine kinase inhibitors do not include PRO assessments [38, 39].

Important International PRO initiatives are ongoing, for example, the standardisation of statistical analyses of PRO data in clinical trials [40]. Also, an international, consensus-based, PRO-specific guidance, the Standard Protocol Items: Recommendations for Interventional Trials (SPIRIT)-PRO Extension, was recently made available [3]. This guidance aims to support investigators with protocol writing and to ensure that all methodological issues are appropriately considered. Finally, the CONSORT PRO Extension has been published in 2013 and this is particularly helpful to investigators at the time of publishing final results of RCTs with a PRO component [35]. Taken together these recommendations will hopefully help investigators improving the design of clinical trials and the assessment of PROs, thus ensuring high-quality data that may inform patient-centred care. Furthermore, it is worth highlighting that the European Organisation for Research and Treatment of Cancer (EORTC) Quality of Life Group has developed various tumour and treatment-specific QoL Modules – with several currently in development, including specific ones for non-muscle invasive BC, muscle invasive BC, and metastatic bladder cancer [41]. Finally, it is important to note, however, that word limits in journal guidelines may sometimes limit authors in the opportunity to report on secondary outcomes (i.e. PROs) for their trials [42] – especially if the results for the primary outcome are negative.

This study has limitations. First, despite our comprehensive search strategy, it is possible that some RCTs with a PRO component might have been missed. Another limitation is the exclusion of non–English language papers. However, it is unlikely that such omission would have significantly altered the conclusion of this review [43]. In addition, we did not compare the published RCT results with their respective protocols, although this might have provided further information. Finally, our results cannot be generalised to RCTs investigating non-conventional medical interventions. A strength of the current review is that we used a formal, objective approach to evaluate PRO reporting in the bladder cancer literature. Since all studies use different reporting criteria and methods, the information was extracted and assessed by two independent researchers. In case of inconsistencies, a third arbiter helped achieving consensus.

## Conclusion

The current systematic review identified little improvement in the uptake and assessment of PROs in RCTs for bladder cancer during the last 4 years. Therefore, given the scarcity of rigorous PRO data, it is difficult to draw meaningful conclusions that can robustly inform patient care and support clinical decision-making. Given the increase in (immunotherapy) drug trials with a potential for severe adverse events in bladder cancer patients, there is urgent need to adopt the recommendations and standards available for PRO use in bladder cancer RCTs.

## Data Availability

All data was taken from publicly available resources and collated in the PROMOTION Registry. Data can be obtained on request: f.efficace@gimema.it.
